# MUA with PRP and corticosteroid injection versus arthroscopic capsular release for refractory frozen shoulder: a retrospective cohort study

**DOI:** 10.3389/fmed.2026.1957034

**Published:** 2026-10-21

**Authors:** Canming Ye, Lunzhu Ciren, Yang Wang, Liu Yang, Kai Wang, Shaoze Lan, Kenan Sun, Chen Wang, Shenglong Pan, Jun Li

**Affiliations:** 1 Department of Orthopedics, The Second Affiliated Hospital of Anhui Medical University, Hefei, China; 2 Institute of Orthopedics, Research Center for Translational Medicine, The Second Affiliated Hospital of Anhui Medical University, Hefei, China; 3 Department of Orthopedics, Shannan City People’s Hospital, Shannan, China; 4 Anhui Medical University, Hefei, China; 5 Department of Orthopedics, The Fourth People’s Hospital of Lu'an, Lu'an, China; 6 Department of Emergency Medicine, The First Affiliated Hospital of Anhui University of Science and Technology, Huainan, China

**Keywords:** arthroscopic capsular release, corticosteroid injection, frozen shoulder, manipulation under anesthesia, platelet-rich plasma, retrospective cohort

## Abstract

**Background:**

Evidence comparing procedural treatment for refractory frozen shoulder is limited. We compared manipulation under anesthesia plus platelet-rich plasma and corticosteroid injection (MUA + PRP + CS) with arthroscopic capsular release (ACR).

**Materials and methods:**

We reviewed records at two centers, 2019–2024. The 1-month visual analog scale (VAS) contrast was a *post hoc* exploratory comparison of early postoperative pain. Other outcomes were American Shoulder and Elbow Surgeons (ASES) score, Shoulder Pain and Disability Index (SPADI), passive range of motion, satisfaction, serious complications or reinterventions, and the hospital-recorded aggregate treatment amount. Mixed-effects models handled repeated measures, and inverse probability of treatment weighting (IPTW) assessed the consistency of longitudinal patterns.

**Results:**

Of 200 screened records, 137 patients were analyzed (MUA + PRP + CS, *n* = 67; ACR, *n* = 70). At 1 month, VAS was lower after MUA + PRP + CS (adjusted mean difference [MD], −0.57; 95% confidence interval [CI], −0.75 to −0.38; Holm-adjusted *P* < 0.001); ASES and forward flexion were higher, while SPADI was lower. ASES also favored the combined strategy at 3 months (MD, 9.09; 95% CI, 7.97–10.22; Holm-adjusted *P* < 0.001). Early VAS and SPADI differences were small. IPTW was directionally consistent with early findings but did not confirm the small 12-month internal-rotation difference. Satisfaction did not differ significantly (98.5% vs. 90.0%; *P* = 0.063). No serious procedure-related complication or reintervention was identified. The adjusted recorded aggregate treatment amount was lower (MD, −6,903.88 Chinese yuan; 95% CI, −7,196.76 to −6,611.00; *P* < 0.001).

**Conclusions:**

In this cohort, MUA + PRP + CS was associated with favorable early outcome differences and a lower recorded aggregate treatment amount than ACR. The 3-month ASES difference was the clearest potentially meaningful early finding. The clinical importance of the 1-month VAS, ASES, and SPADI differences, as well as the small internal-rotation difference, remains uncertain. Residual confounding precludes causal comparative inferences.

## Highlights

- The combined strategy was associated with favorable early outcome differences.
- The 3-month ASES difference was the most clinically interpretable early signal.
- The 1-month VAS and SPADI differences were statistically significant but small in magnitude.
- Most 12-month comparisons were not statistically significant.
- The hospital-recorded aggregate treatment amount was lower with the combined strategy.

## 1 Introduction

Frozen shoulder (adhesive capsulitis) causes pain and restriction of active and passive glenohumeral motion, affecting sleep, daily activities, work, quality of life, and psychological and social well-being (1–6). Although most patients improve with nonoperative care, persistent symptoms may prompt procedural treatment (1, 3, 4, 7–10).

Manipulation under anesthesia (MUA) and arthroscopic capsular release (ACR) are established options for refractory disease. Randomized and pooled comparisons report improvement after both, without consistent late superiority (2, 7–9, 11–15). MUA can restore motion rapidly with fewer surgical resources, but forceful manipulation risks osseous, soft-tissue, or neurovascular injury (2, 4, 11–13, 16). ACR allows visualized release but requires arthroscopic resources and may add early postoperative pain and treatment burden (3, 8, 9, 11, 17).

The participating centers routinely used either MUA plus platelet-rich plasma (PRP) and corticosteroid injection (CS), referred to here as the combined strategy, or ACR. Manipulation targets adhesions, CS may reduce early inflammation, and PRP has been studied as a biological adjunct (4, 10, 18–28). This rationale does not establish component efficacy; pooled evidence has not shown a consistent benefit from adding intra-articular corticosteroid to MUA (29). Without component-specific comparators, pathway differences cannot be attributed to MUA, PRP, or CS. Evidence comparing the recovery trajectories and hospital-recorded aggregate treatment amounts of these complete strategies remains limited.

We compared these routine pathways over 12 months, examining early pain recovery, function, disability, range of motion, satisfaction, serious complications or reinterventions, and the hospital-recorded aggregate treatment amount. IPTW was used as a sensitivity analysis for longitudinal clinical outcomes. The study was intended to describe recovery patterns in routine care and inform shared decision-making; it cannot establish causality, the effect of individual treatment components, equivalence, or cost-effectiveness. It is reported in line with the STROCSS 2024 guideline (30).

## 2 Materials and methods

### 2.1 Study design and ethics

This two-center retrospective cohort study used clinical records from The Second Affiliated Hospital of Anhui Medical University and Shannan City People's Hospital. We screened patients with refractory frozen shoulder who received routine care with MUA plus PRP and CS or ACR between January 2019 and December 2024. All procedures were delivered as usual care rather than for research.

The retrospective analysis protocol was approved by the Medical Ethics Committee of Shannan City People's Hospital on June 8, 2026, after completion of the clinical care period (approval number 20260608002). The committee waived written informed consent for analysis of anonymized records. The analysis followed the Declaration of Helsinki.

### 2.2 Participants

Patients were identified in institutional medical record systems using diagnostic and procedure codes. Frozen shoulder was diagnosed from symptoms, physical examination, and magnetic resonance imaging (MRI), following accepted diagnostic principles (1, 3, 4). Diagnosis required persistent pain with restricted active and passive glenohumeral motion, particularly forward flexion, abduction, external rotation, and internal rotation. Preoperative MRI was used to confirm the diagnosis and exclude structural disorders that could affect function, including full-thickness rotator cuff tears, severe cuff arthropathy, advanced glenohumeral osteoarthritis, tumors, and other relevant intra-articular lesions.

The final cohort comprised 137 patients: 67 received MUA plus PRP and CS (the combined-strategy group), and 70 underwent ACR (the ACR group). Extracted baseline variables included age, sex, symptom duration, dominant-side involvement, diabetes mellitus, study site, and the baseline value of each clinical outcome.

### 2.3 Inclusion and exclusion criteria

Eligibility required age of at least 18 years, a clinical diagnosis of frozen shoulder, persistent pain and stiffness despite at least 3 months of conservative treatment, treatment with MUA plus PRP and CS or ACR, records sufficient for outcome analysis, and at least 12 months of follow-up.

Records were excluded when the diagnosis was not confirmed, a full-thickness rotator cuff tear was present, the affected shoulder had undergone previous surgery, follow-up was shorter than 12 months, or records were insufficient for analysis. Additional exclusions were structural shoulder disorders, previous fracture or dislocation, infection, tumor, and neurologic conditions affecting the shoulder.

### 2.4 Treatment allocation

Treatment followed routine practice rather than random assignment. Surgeons and patients selected a strategy through shared decision-making about expected benefits, limitations, invasiveness, recovery, safety, treatment burden, and financial considerations. Clinical presentation, symptom duration, MRI findings, patient preference, and economic circumstances informed the choice.

Patients seeking a less invasive approach generally chose the combined MUA strategy; those preferring arthroscopy or requiring direct capsular visualization generally underwent ACR. Baseline characteristics were therefore compared before outcome modeling.

### 2.5 Interventions

#### 2.5.1 MUA plus PRP and CS

Experienced shoulder surgeons performed all combined-strategy procedures under general anesthesia with the patient supine. The scapula was stabilized against the thorax after adequate muscle relaxation. Manipulation proceeded through forward elevation, abduction, and external and internal rotation at 0, 45, and 90 degrees of abduction. A gradual short-lever-arm technique was used to limit fracture, dislocation, rotator cuff injury, and neurovascular injury. Passive motion was reassessed immediately afterwards.

Autologous PRP was prepared with the RegenKit BCT system (Regen Lab SA, Switzerland) according to the manufacturer's instructions. Approximately 8 mL of peripheral venous blood was collected into a sterile tube containing sodium citrate and thixotropic separating gel. After centrifugation at 1,500 × g for 5 min, 4–5 mL of leukocyte-reduced PRP was collected under sterile conditions, consistent with reported intra-articular protocols (18–21, 31).

After manipulation, the PRP was injected into the glenohumeral joint through a posterior landmark-guided approach under sterile conditions. The corticosteroid injection contained 40 mg of triamcinolone acetonide, a dose used in published intra-articular protocols for frozen shoulder (32, 33). The available procedural summary did not specify whether CS was mixed with PRP or injected separately, or record the final injectate volume.

#### 2.5.2 Arthroscopic capsular release

Patients in the ACR group received general anesthesia. Passive shoulder motion was assessed before the patient was placed in the lateral decubitus position. Standard posterior viewing and anterior working portals were established. Diagnostic arthroscopy evaluated capsular thickening, synovial hypertrophy, rotator interval contracture, and associated intra-articular lesions.

Capsular release used radiofrequency ablation and an arthroscopic shaver. Synovial hypertrophy was debrided when present. Release proceeded from the rotator interval and coracohumeral ligament to the anterior and inferior capsule. The anterior and posterior bands of the inferior glenohumeral ligament were released while protecting the axillary nerve. Posterior release was added when posterior contracture was identified. Passive motion was reassessed after release. No additional procedure was performed unless arthroscopy identified a lesion requiring treatment.

### 2.6 Postoperative rehabilitation

Both groups followed the same rehabilitation principles under the treating surgeon and rehabilitation team. Exercises began after recovery from anesthesia when pain was tolerable. Initial therapy emphasized passive and assisted active forward flexion, abduction, external rotation, and internal rotation. Patients performed stretching several times daily to maintain the motion gained during the procedure.

During the first postoperative weeks, rehabilitation focused on pain control and prevention of recurrent stiffness. Active motion and strengthening of the rotator cuff and periscapular muscles were added as symptoms and mobility improved. Exercise intensity was adjusted to pain tolerance, while follow-up schedules and rehabilitation principles remained consistent between groups.

### 2.7 Outcome measures

Outcomes were assessed before treatment and at 1, 3, 6, and 12 months. Because the study was not prospectively registered, no outcome was prespecified. After data collection, the adjusted between-group VAS comparison at 1 month was highlighted as an exploratory description of early postoperative pain. It was not a primary outcome or a confirmatory measure of early recovery. Pain was highlighted because it is patient reported and may affect early rehabilitation; range of motion (ROM), ASES, and SPADI were coequal exploratory outcomes assessed at the same time points. Pain was measured with the visual analog scale (VAS), where higher scores indicate greater pain (34). Shoulder function was measured with the American Shoulder and Elbow Surgeons (ASES) score, where higher scores indicate better function (35). The Shoulder Pain and Disability Index (SPADI) assessed pain-related disability, with higher scores indicating worse status (36).

Trained clinicians measured passive forward flexion, abduction, external rotation, and internal rotation in degrees using the centers' standardized clinical method. Passive external rotation was measured with the shoulder in 0 degrees of abduction (upper arm at the side), the elbow flexed to 90 degrees, and the scapula stabilized. Satisfaction at final follow-up was classified as satisfied or not satisfied.

The safety analysis was limited to serious procedure-related complications and reinterventions identifiable from medical records, operative reports, follow-up notes, and clinically indicated imaging. These outcomes included humeral fracture, shoulder dislocation, rotator cuff injury, neurovascular injury, infection, persistent pain or recurrent stiffness requiring another intervention, reoperation, and any secondary procedure. The available aggregate data did not support systematic classification of mild or transient events, including pain flares or paresthesia.

### 2.8 Statistical analysis

Distributions were examined with histograms, Q-Q plots, and the Shapiro–Wilk test when appropriate. Continuous variables were reported as mean [standard deviation (SD)] or median (interquartile range), and categorical variables as number (%). Baseline comparisons used independent-samples t tests, Mann–Whitney U tests, chi-square tests, or Fisher exact tests, as appropriate. Absolute standardized mean differences (SMDs) described balance; values below 0.10 indicated good balance.

All longitudinal clinical outcomes were analyzed at 1, 3, 6, and 12 months. The 1-month VAS contrast was highlighted after data collection as an exploratory early postoperative pain analysis, rather than as a prespecified or confirmatory early-recovery outcome. Other outcomes were ASES, SPADI, passive range of motion, satisfaction, serious complications or reinterventions, and the hospital-recorded aggregate treatment amount. Linear mixed-effects models accounted for repeated measurements and used all available observations (37). The repeated response comprised postoperative observations at 1, 3, 6, and 12 months; the corresponding baseline outcome entered the model only as a covariate. Separate models were fitted for each longitudinal outcome. Fixed effects included group, time, and their interaction, with a patient-level random intercept. Models adjusted for the corresponding baseline outcome, age, sex, dominant-side involvement, symptom duration, diabetes mellitus, and study site. Mean differences (MDs) were calculated as the combined-strategy group minus the ACR group and reported with 95% confidence intervals (CIs). The four postoperative comparisons within each outcome formed the family for Holm adjustment (38).

We used IPTW as a sensitivity analysis for nonrandom treatment allocation (39). The propensity model included age, sex, dominant-side involvement, symptom duration, diabetes mellitus, study site, and all baseline clinical outcomes. Conventional logistic estimation showed near-complete separation with these prespecified covariates, so a ridge-penalized logistic model was used to stabilize propensity estimates. Continuous covariates were standardized before fitting, and the L2 penalty parameter was set to 0.01 from balance diagnostics without reference to outcome data. Stabilized weights were truncated at the 2.5th and 97.5th percentiles. Balance was assessed with absolute SMDs; values below 0.10 were considered acceptable (40). Weighted generalized estimating equations with an exchangeable working correlation and robust standard errors repeated the longitudinal comparisons. We examined weight distributions, effective sample size, and post-weighting balance. These analyses were limited to longitudinal clinical outcomes and were interpreted as sensitivity analyses; they were not applied to the recorded treatment amount or safety outcomes.

Only the hospital-recorded aggregate treatment amount was available. Itemized components, the billing window, recording basis, and price-standardization procedures were undocumented. We therefore analyzed the recorded aggregate amount without treating it as a direct medical cost, component cost, patient expenditure, or standardized measure of resource use. Unadjusted and adjusted linear regression were used. The adjusted model included age, sex, dominant-side involvement, symptom duration, diabetes mellitus, and study site, with HC3 heteroskedasticity-consistent standard errors. Satisfaction was compared with Fisher exact test and reported as an odds ratio (OR) with a 95% CI. Serious complications and reinterventions were summarized with exact binomial upper 95% confidence limits. Tests were two-sided. Analyses used Python 3.12, statsmodels 0.14.6, and SciPy 1.18.0.

No *a priori* sample-size calculation was performed because all eligible records from the study period were included. An observed-effect *post hoc* power calculation was not performed; effect estimates and 95% CIs were used to describe precision. Mixed models used available observations without imputation and assumed data were missing at random. One ACR patient lacked four postoperative abduction measurements; the reason was not documented in the available analysis materials, and the missing-at-random assumption could not be empirically verified. All other longitudinal outcomes were complete. All outcome analyses were exploratory.

## 3 Results

### 3.1 Patient selection and baseline characteristics

Of 200 screened records, 63 were excluded: 15 did not meet diagnostic criteria, 12 had a full-thickness rotator cuff tear, 10 had previous shoulder surgery, 18 had less than 12 months of follow-up, and 8 had records insufficient for analysis. The complete-follow-up cohort comprised 137 patients: 67 in the combined-strategy group and 70 in the ACR group (Figure 1).

**Figure 1:**
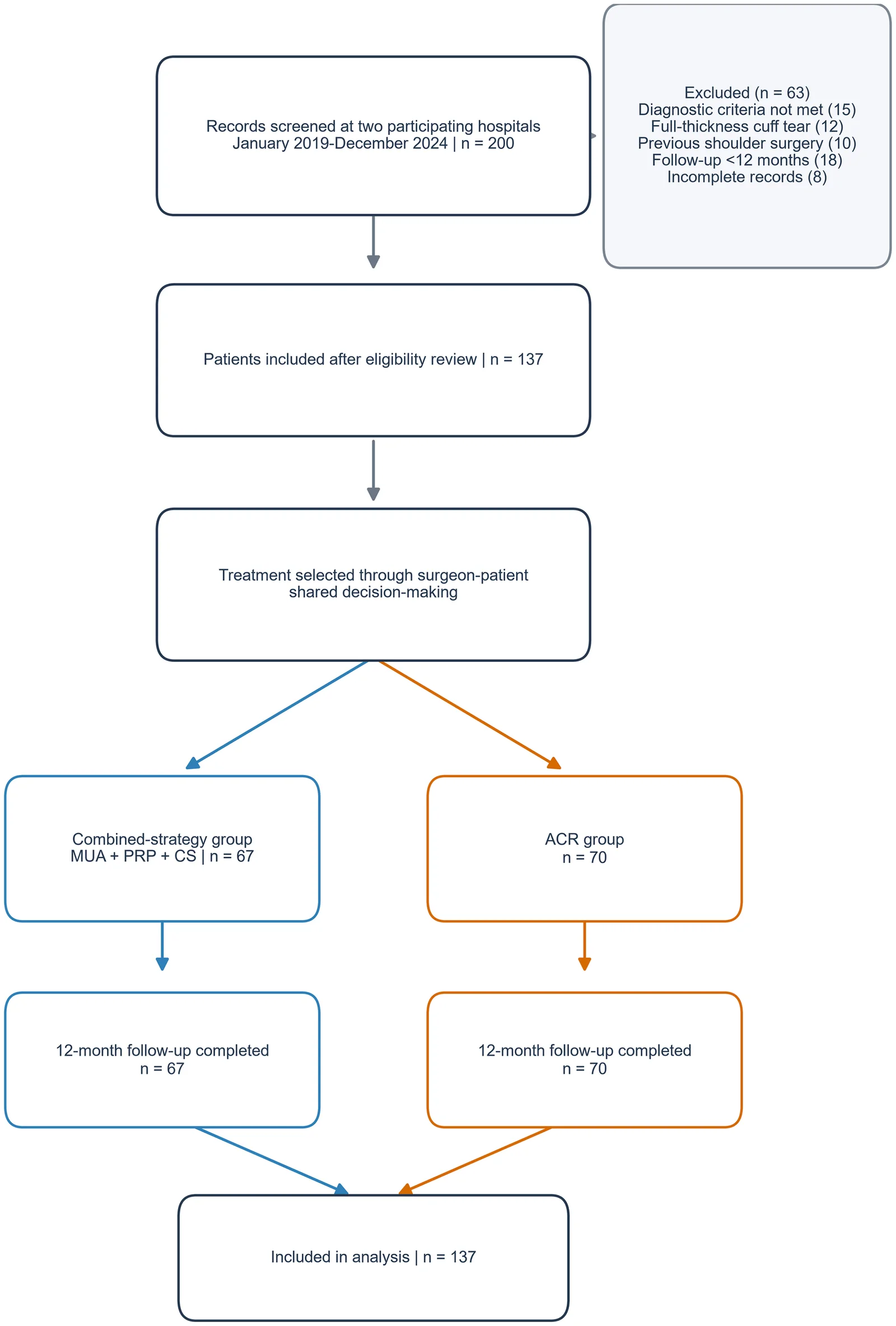
Patient selection and treatment allocation. Records from both hospitals were screened; 63 of 200 were excluded for the reasons shown, leaving 137 patients for analysis. Treatment followed surgeon-patient shared decision-making rather than random allocation. ACR, arthroscopic capsular release; CS, corticosteroid injection; MUA, manipulation under anesthesia; PRP, platelet-rich plasma.

Table 1 summarizes baseline characteristics. Each group included 17 patients from Shannan City People's Hospital. Diabetes mellitus was present in 23 of 67 (34.3%) and 21 of 70 (30.0%) patients, respectively. The largest imbalance was baseline ASES score (absolute SMD, 0.29); site and diabetes were closely balanced (absolute SMDs, 0.03 and 0.09).

**Table 1:** Baseline demographic and clinical characteristics.

Variable	Combined-strategy group (*n* = 67)	ACR group (*n* = 70)	Abs. SMD	*P* value
Age, years	57.72 ± 7.66	56.66 ± 9.86	0.12	0.485
Male sex, *n* (%)	26 (38.8)	24 (34.3)	0.09	0.583
Symptom duration, months	10.06 ± 5.94	8.91 ± 5.47	0.20	0.242
Dominant-side involvement, yes, *n* (%)	51 (76.1)	59 (84.3)	0.21	0.230
Diabetes mellitus, yes, *n* (%)	23 (34.3)	21 (30.0)	0.09	0.588
Shannan City People's Hospital, *n* (%)	17 (25.4)	17 (24.3)	0.03	0.883
Preoperative VAS score	8.13 ± 0.76	8.17 ± 0.72	0.05	0.769
Preoperative ASES score	42.69 ± 4.06	41.40 ± 4.74	0.29	0.091
Preoperative SPADI score	86.18 ± 3.14	86.90 ± 3.29	0.22	0.192
Preoperative forward flexion, degrees	76.87 ± 13.37	76.29 ± 13.74	0.04	0.803
Preoperative abduction, degrees	77.01 ± 13.20	76.07 ± 13.64	0.07	0.682
Preoperative external rotation, degrees	32.01 ± 6.69	33.36 ± 6.35	0.21	0.231
Preoperative internal rotation, degrees	36.94 ± 6.74	38.36 ± 6.35	0.22	0.208

Data are mean (SD) or *n* (%); absolute SMDs describe baseline balance. ACR, arthroscopic capsular release; ASES, American Shoulder and Elbow Surgeons; CS, corticosteroid injection; MUA, manipulation under anesthesia; PRP, platelet-rich plasma; SD, standard deviation; SMD, standardized mean difference; SPADI, Shoulder Pain and Disability Index; VAS, visual analog scale.

### 3.2 Early patient-reported outcomes

VAS improved in both groups, with a group-by-time interaction (*P* < 0.001). At 1 month, VAS was lower in the combined-strategy group (adjusted MD, −0.57; 95% CI, −0.75 to −0.38; Holm-adjusted *P* < 0.001). No significant between-group difference was detected at later follow-up visits.

ASES was higher in the combined-strategy group at 1 month (adjusted MD, 2.00; 95% CI, 0.88–3.12; Holm-adjusted *P* = 0.001) and 3 months (adjusted MD, 9.09; 95% CI, 7.97–10.22; Holm-adjusted *P* < 0.001), while SPADI was lower at 1 month (adjusted MD, −2.66; 95% CI, −3.44 to −1.89; Holm-adjusted *P* < 0.001). Later comparisons were not significant.

Contextual reference values were examined to aid interpretation. In a separate cohort of nondiabetic patients undergoing ACR, distribution-based within-patient minimal clinically important differences (MCIDs) at 6 months were 1.1 points for VAS and 8.2 points for ASES (41). These values are not validated between-group thresholds at 1 or 3 months in the present cohort. The 1-month VAS and ASES estimates (−0.57 and 2.00 points) were smaller than those contextual values, whereas the 3-month ASES estimate (9.09 points) was numerically larger. In adhesive capsulitis, a 17-point SPADI smallest detectable difference has been reported (42); the 1-month SPADI estimate (−2.66 points) was smaller.

Table 2 and Figure 2 show the complete trajectories.

**Table 2:** Longitudinal changes in pain, shoulder function, and disability.

Outcome	Time point	Combined-strategy group	ACR group	Adjusted MD (95% CI)	Holm-adjusted *P* value
VAS score	Preoperative	8.13 ± 0.76	8.17 ± 0.72	−0.04 (−0.28 to 0.21)	0.769
	1 month	4.18 ± 0.69	4.76 ± 0.75	−0.57 (−0.75 to −0.38)	<0.001
	3 months	2.33 ± 0.66	2.21 ± 0.56	0.12 (−0.06 to 0.31)	0.580
	6 months	1.12 ± 0.37	1.07 ± 0.26	0.06 (−0.13 to 0.24)	1.000
	12 months	0.40 ± 0.49	0.40 ± 0.49	0.01 (−0.17 to 0.20)	1.000
ASES score	Preoperative	42.69 ± 4.06	41.40 ± 4.74	1.29 (−0.19 to 2.76)	0.091
	1 month	56.04 ± 4.25	53.87 ± 3.39	2.00 (0.88 to 3.12)	0.001
	3 months	66.19 ± 4.16	56.93 ± 3.30	9.09 (7.97 to 10.22)	<0.001
	6 months	86.99 ± 3.01	87.10 ± 3.63	−0.29 (−1.41 to 0.84)	0.615
	12 months	92.72 ± 2.37	93.21 ± 2.38	−0.67 (−1.80 to 0.45)	0.483
SPADI score	Preoperative	86.18 ± 3.14	86.90 ± 3.29	−0.72 (−1.80 to 0.36)	0.192
	1 month	68.34 ± 2.62	71.04 ± 3.28	−2.66 (−3.44 to −1.89)	<0.001
	3 months	51.21 ± 1.19	51.20 ± 1.08	0.05 (−0.73 to 0.82)	1.000
	6 months	32.78 ± 1.18	32.80 ± 1.15	0.01 (−0.76 to 0.79)	1.000
	12 months	14.42 ± 3.15	14.16 ± 3.27	0.30 (−0.48 to 1.07)	1.000

Data are mean (SD) unless indicated otherwise. Follow-up MDs came from baseline-adjusted mixed-effects models and were calculated as the combined-strategy group minus the ACR group. Baseline rows show unadjusted MDs. Follow-up *P* values were Holm-adjusted within each outcome. Lower VAS and SPADI scores and higher ASES scores indicate better outcomes. The combined-strategy group received MUA plus PRP and CS. ACR, arthroscopic capsular release; ASES, American Shoulder and Elbow Surgeons; CI, confidence interval; CS, corticosteroid injection; MD, mean difference; MUA, manipulation under anesthesia; PRP, platelet-rich plasma; SD, standard deviation; SPADI, Shoulder Pain and Disability Index; VAS, visual analog scale.

**Figure 2:**
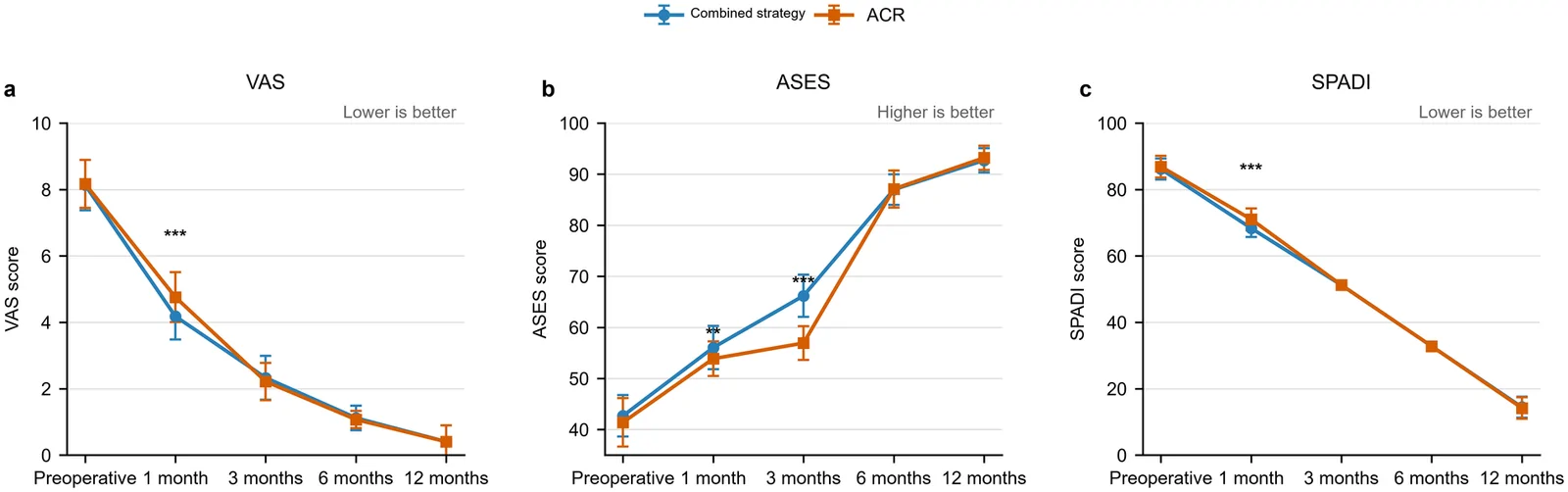
Longitudinal patient-reported outcomes: **(a)** VAS, **(b)** ASES, and **(c)** SPADI. Points and error bars show unadjusted mean (SD) before treatment and at 1, 3, 6, and 12 months. Asterisks denote Holm-adjusted between-group comparisons from baseline-adjusted mixed-effects models. The combined-strategy group had lower VAS and SPADI at 1 month and higher ASES at 1 and 3 months; later comparisons were nonsignificant. ACR, arthroscopic capsular release; ASES, American Shoulder and Elbow Surgeons; CS, corticosteroid injection; MUA, manipulation under anesthesia; PRP, platelet-rich plasma; SPADI, Shoulder Pain and Disability Index; SD, standard deviation; VAS, visual analog scale. Double and triple asterisks indicate *P* < 0.01 and *P* < 0.001, respectively.

### 3.3 Passive range-of-motion outcomes

Passive forward flexion showed a group-by-time interaction (*P* < 0.001) and was greater in the combined-strategy group at 1 month (adjusted MD, 8.31 degrees; 95% CI, 5.52 to 11.10; Holm-adjusted *P* < 0.001). No significant between-group difference was detected thereafter.

Abduction and external rotation did not differ significantly at follow-up. At 12 months, internal rotation favored the combined-strategy group in the main model (adjusted MD, 2.10 degrees; 95% CI, 1.01–3.20; Holm-adjusted *P* < 0.001). This small difference was not confirmed by IPTW (weighted MD, 2.03 degrees; 95% CI, 0.02–4.03; Holm-adjusted *P* = 0.193) and was interpreted cautiously (Table 3; Figure 3; Supplementary Table S2).

**Table 3:** Longitudinal changes in passive shoulder range of motion.

Outcome	Time point	Combined-strategy group	ACR group	Adjusted MD (95% CI)	Holm-adjusted *P* value
Forward flexion, degrees	Preoperative	76.87 ± 13.37	76.29 ± 13.74	0.58 (−3.96 to 5.12)	0.803
	1 month	154.78 ± 13.10	146.36 ± 12.42	8.31 (5.52 to 11.10)	<0.001
	3 months	162.54 ± 10.49	162.64 ± 5.94	−0.21 (−3.00 to 2.57)	1.000
	6 months	170.97 ± 8.31	172.36 ± 3.78	−1.49 (−4.28 to 1.29)	0.880
	12 months	175.22 ± 4.96	175.36 ± 4.28	−0.24 (−3.03 to 2.55)	1.000
Abduction, degrees	Preoperative	77.01 ± 13.20	76.07 ± 13.64	0.94 (−3.55 to 5.44)	0.682
	1 month	117.76 ± 8.27	118.04 ± 8.41	−0.63 (−3.18 to 1.91)	1.000
	3 months	125.67 ± 6.51	125.65 ± 6.41	−0.33 (−2.88 to 2.21)	1.000
	6 months	136.42 ± 7.22	135.87 ± 7.52	0.20 (−2.35 to 2.74)	1.000
	12 months	148.13 ± 7.97	147.83 ± 8.38	−0.04 (−2.59 to 2.50)	1.000
External rotation, degrees	Preoperative	32.01 ± 6.69	33.36 ± 6.35	−1.34 (−3.53 to 0.84)	0.231
	1 month	44.48 ± 5.37	44.64 ± 5.20	0.57 (−0.28 to 1.42)	0.749
	3 months	51.49 ± 3.14	51.71 ± 2.68	0.51 (−0.33 to 1.36)	0.749
	6 months	60.52 ± 4.62	60.79 ± 3.57	0.47 (−0.37 to 1.32)	0.749
	12 months	65.52 ± 4.78	65.79 ± 3.67	0.47 (−0.37 to 1.32)	0.749
Internal rotation, degrees	Preoperative	36.94 ± 6.74	38.36 ± 6.35	−1.42 (−3.61 to 0.78)	0.208
	1 month	50.30 ± 5.46	50.64 ± 5.20	0.38 (−0.72 to 1.47)	1.000
	3 months	56.57 ± 3.28	57.07 ± 2.76	0.22 (−0.88 to 1.31)	1.000
	6 months	66.49 ± 3.99	66.07 ± 3.80	1.14 (0.05 to 2.23)	0.122
	12 months	75.24 ± 6.11	73.86 ± 5.37	2.10 (1.01 to 3.20)	<0.001†

Data are mean (SD). Follow-up MDs (combined-strategy group minus ACR group) came from mixed-effects models adjusted for the baseline outcome, age, sex, dominant-side involvement, symptom duration, diabetes mellitus, and study site; *P* values were Holm-adjusted within each outcome. Postoperative abduction data were available for 67 and 69 patients, respectively. One patient in the ACR group had missing postoperative abduction data; the reason was not documented. †The 12-month internal-rotation difference was significant in the primary mixed-effects model but was not confirmed in the IPTW sensitivity analysis (weighted MD, 2.03 degrees; 95% CI, 0.02 to 4.03; Holm-adjusted *P* = 0.193). The combined-strategy group received MUA plus PRP and CS. ACR, arthroscopic capsular release; CI, confidence interval; CS, corticosteroid injection; IPTW, inverse probability of treatment weighting; MD, mean difference; MUA, manipulation under anesthesia; PRP, platelet-rich plasma; SD, standard deviation.

**Figure 3:**
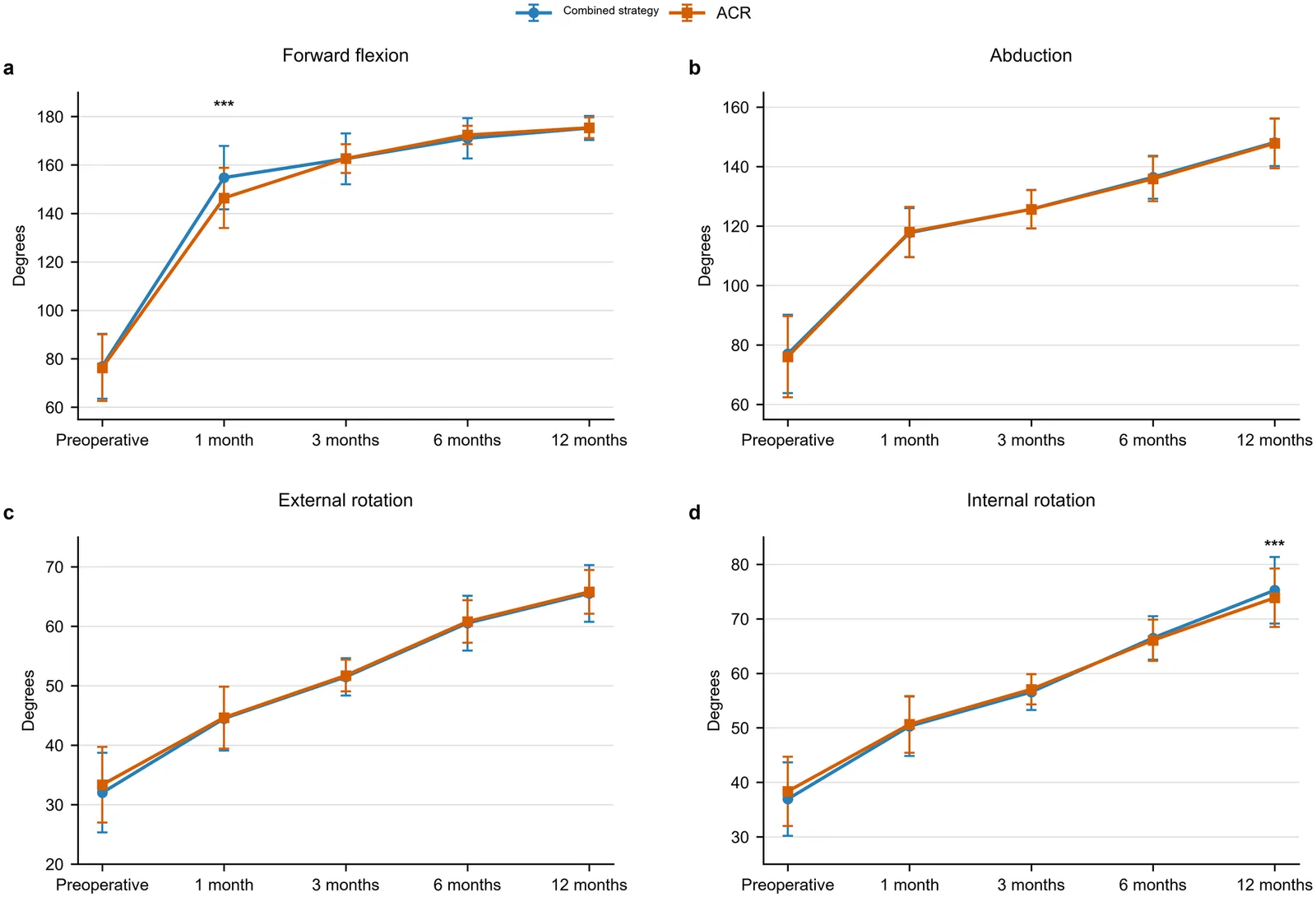
Longitudinal passive shoulder range of motion: **(a)** forward flexion, **(b)** abduction, **(c)** external rotation, and **(d)** internal rotation. Points and error bars show unadjusted mean (SD) before treatment and at 1, 3, 6, and 12 months. Asterisks denote Holm-adjusted comparisons from covariate-adjusted mixed-effects models. Passive forward flexion favored the combined-strategy group at 1 month. The small 12-month passive internal-rotation difference from the primary model was not confirmed by IPTW. ACR, arthroscopic capsular release; CS, corticosteroid injection; IPTW, inverse probability of treatment weighting; MUA, manipulation under anesthesia; PRP, platelet-rich plasma; SD, standard deviation. Triple asterisks indicate *P* < 0.001.

### 3.4 Twelve-month outcomes and satisfaction

At 12 months, no statistically significant between-group difference was detected in VAS, ASES, SPADI, forward flexion, abduction, or external rotation. The primary model showed a small internal-rotation difference favoring the combined-strategy group (adjusted MD, 2.10 degrees; 95% CI, 1.01–3.20; Holm-adjusted *P* < 0.001), but this result was not confirmed by IPTW (weighted MD, 2.03 degrees; 95% CI, 0.02–4.03; Holm-adjusted *P* = 0.193) (Table 4; Figure 4; Supplementary Table S2).

**Table 4:** Twelve-month outcomes, satisfaction, recorded serious safety outcomes, and hospital-recorded aggregate treatment amount.

Variable	Combined-strategy group (*n* = 67)	ACR group (*n* = 70)	Effect estimate (95% CI)	*P* value
12-month VAS score	0.40 ± 0.49	0.40 ± 0.49	0.01 (−0.17 to 0.20)	1.000
12-month ASES score	92.72 ± 2.37	93.21 ± 2.38	−0.67 (−1.80 to 0.45)	0.483
12-month SPADI score	14.42 ± 3.15	14.16 ± 3.27	0.30 (−0.48 to 1.07)	1.000
12-month forward flexion, degrees	175.22 ± 4.96	175.36 ± 4.28	−0.24 (−3.03 to 2.55)	1.000
12-month abduction, degrees	148.13 ± 7.97	147.83 ± 8.38	−0.04 (−2.59 to 2.50)	1.000
12-month external rotation, degrees	65.52 ± 4.78	65.79 ± 3.67	0.47 (−0.37 to 1.32)	0.749
12-month internal rotation, degrees	75.24 ± 6.11	73.86 ± 5.37	2.10 (1.01 to 3.20)	<0.001†
Patient satisfaction, *n* (%)	66 (98.5)	63 (90.0)	OR 7.33 (0.88 to 61.31)	0.063
Recorded serious procedure-related complications or reinterventions, *n* (%)	0 (0.0)	0 (0.0)	Exact upper 95% CI: 5.36% vs 5.13%	NA
Hospital-recorded aggregate treatment amount, CNY	7,975.49 ± 431.77	14,897.57 ± 1,119.07	−6,903.88 (−7,196.76 to −6,611.00)	<0.001

Data are mean (SD) or *n* (%) unless indicated otherwise. The combined-strategy group received MUA plus PRP and CS. Clinical estimates came from covariate-adjusted mixed-effects models and were calculated as the combined-strategy group minus the ACR group. Satisfaction is reported as an odds ratio. *P* values for 12-month clinical outcomes were Holm-adjusted within their longitudinal outcome families. Recorded aggregate treatment amount was adjusted for age, sex, dominant-side involvement, symptom duration, diabetes mellitus, and study site. Exact upper 95% CI bounds are shown for zero serious events. †The 12-month internal-rotation primary-model finding was not confirmed by IPTW (weighted MD, 2.03 degrees; 95% CI, 0.02 to 4.03; Holm-adjusted *P* = 0.193). ACR, arthroscopic capsular release; ASES, American Shoulder and Elbow Surgeons; CI, confidence interval; CNY, Chinese yuan; CS, corticosteroid injection; IPTW, inverse probability of treatment weighting; MUA, manipulation under anesthesia; NA, not applicable; OR, odds ratio; PRP, platelet-rich plasma; SD, standard deviation; SPADI, Shoulder Pain and Disability Index; VAS, visual analog scale.

**Figure 4:**
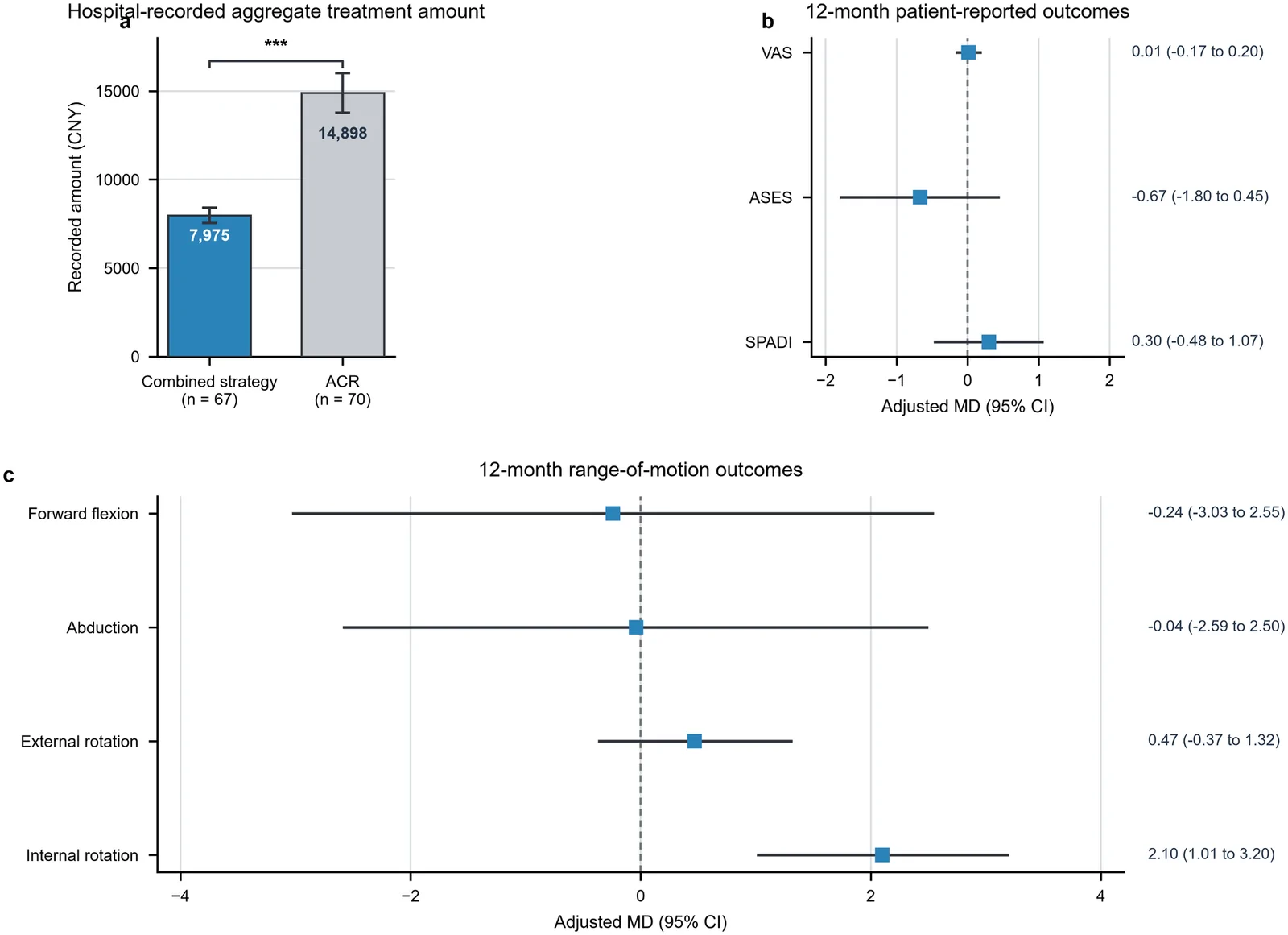
Hospital-recorded aggregate treatment amount and adjusted 12-month differences. **(a)** Group means for the recorded aggregate treatment amount (error bars, SD). **(b)** Adjusted MDs in patient-reported outcomes. **(c)** Adjusted MDs in passive range of motion. MDs are the combined-strategy group minus the ACR group; error bars in panels b and c show 95% CIs. Most clinical intervals crossed zero. Although the primary-model passive internal-rotation interval did not cross zero, the finding was not confirmed by IPTW. The panel-a recorded-amount comparison was significant in the adjusted linear model (*P* < 0.001); its error bars show SD rather than CIs. ACR, arthroscopic capsular release; ASES, American Shoulder and Elbow Surgeons; CI, confidence interval; CNY, Chinese yuan; IPTW, inverse probability of treatment weighting; MD, mean difference; SD, standard deviation; SPADI, Shoulder Pain and Disability Index; VAS, visual analog scale. Triple asterisks indicate *P* < 0.001 for the adjusted recorded-amount model.

Satisfaction was reported by 66 of 67 patients (98.5%) in the combined-strategy group and 63 of 70 (90.0%) in the ACR group. Groups did not differ significantly (OR, 7.33; 95% CI, 0.88–61.31; Fisher *P* = 0.063).

### 3.5 Hospital-recorded aggregate treatment amount

The mean (SD) hospital-recorded aggregate treatment amount was 7,975.49 (431.77) Chinese yuan (CNY) in the combined-strategy group and 14,897.57 (1,119.07) CNY in the ACR group. The unadjusted MD was −6,922.08 CNY; the adjusted MD was −6,903.88 CNY (95% CI, −7,196.76 to −6,611.00; *P* < 0.001). This aggregate amount was not a fully specified direct medical cost or a formal economic evaluation.

### 3.6 IPTW sensitivity analysis and safety outcomes

IPTW improved covariate balance (Supplementary Table S1; Supplementary Figure S1). Before truncation, stabilized weights ranged from 0.52 to 4.86; after truncation, they ranged from 0.58 to 1.53. The effective sample size was 128.5, and all post-weighting absolute SMDs were below 0.10 (maximum, 0.097). Supplementary Table S2 reports weighted estimates for every postoperative outcome and time point. Weighted analyses were directionally consistent with the early VAS, ASES, SPADI, and forward-flexion findings. However, the 12-month internal-rotation difference did not remain significant after Holm adjustment (weighted MD, 2.03 degrees; 95% CI, 0.02–4.03; Holm-adjusted *P* = 0.193). No serious procedure-related complication or reintervention was identified. Mild or transient events were not systematically captured. Exact upper 95% confidence limits for zero events were 5.36% and 5.13%.

## 4 Discussion

### 4.1 Principal findings

Early between-group outcome differences favored the combined strategy, but their magnitude and clinical relevance varied by outcome. The 3-month ASES difference was the clearest potentially meaningful signal. The 1-month VAS, ASES, and SPADI differences were statistically significant but small when interpreted against the available contextual references. Most 12-month comparisons were not statistically significant. The small passive internal-rotation difference in the primary model was not confirmed by IPTW. The hospital-recorded aggregate treatment amount was lower in the combined-strategy group. No serious procedure-related complication or reintervention was identified in available records.

These results extend, rather than overturn, evidence that MUA and ACR both improve pain and function without consistent late superiority (2, 7–9, 11–15). This two-center routine-practice comparison describes recovery trajectories and the hospital-recorded aggregate treatment amount for complete treatment strategies. It cannot distinguish the contribution of individual treatment components.

### 4.2 Early recovery and component uncertainty

Manipulation may disrupt adhesions and restore passive motion, while corticosteroid may reduce early inflammation and improve rehabilitation tolerance. Any biological contribution of PRP cannot be isolated in this cohort. Pooled evidence has not shown a consistent advantage from adding corticosteroid to MUA (29). Without MUA-only, PRP-only, or CS-only comparators, no early difference can be attributed to a single component.

The 3-month ASES difference was the clearest potentially meaningful early result. In a separate cohort of nondiabetic patients undergoing ACR, distribution-based within-patient MCIDs at 6 months were 1.1 points for VAS and 8.2 points for ASES (41). The 1-month VAS and ASES estimates (−0.57 and 2.00 points) were smaller than these contextual values, whereas the 3-month ASES estimate (9.09 points) was numerically larger. These values arose from a different population, time point, and within-patient construct; they are not validated between-group thresholds for this cohort. A 17-point SPADI smallest detectable difference, rather than an MCID, has been reported in adhesive capsulitis (42), and the 1-month SPADI estimate (−2.66 points) was smaller. Statistical significance alone therefore does not establish the clinical importance of these early estimates.

ACR permits controlled visualized release but may add arthroscopic tissue trauma and postoperative irritation. Whether this explains the early trajectory cannot be determined here. ACR patients also improved substantially, and the pattern should not be interpreted as inferior ACR efficacy.

### 4.3 Twelve-month outcomes and clinical choice

Most 12-month comparisons were not statistically significant. The main model showed a 2.10-degree internal-rotation difference, but the IPTW analysis did not confirm it after Holm adjustment. Because the analysis used a superiority framework without an equivalence or noninferiority margin, negative tests cannot establish equivalent or noninferior effects.

The internal-rotation finding remains exploratory. MUA may produce broader passive capsular stretching, whereas ACR provides visualized, selective release, with posterior release added only when posterior contracture is identified. This explanation was not tested. A validated disease-specific MCID for internal rotation was not available, and a 2.10-degree estimate may lie within measurement variation; its clinical importance is therefore uncertain.

Treatment choice remains individualized. Earlier recovery and a lower recorded aggregate treatment amount may matter to some patients, whereas direct visualization may favor ACR when intra-articular pathology is suspected. Rehabilitation tolerance, comorbidity, local resources, financial circumstances, and patient preference remain relevant.

### 4.4 Safety considerations

MUA is less invasive than arthroscopy but can cause fracture, dislocation, rotator cuff or labral injury, and neurovascular damage, particularly with excessive force or a long lever arm (2, 11, 12, 16). The combined-strategy procedures used scapular stabilization and gradual short-lever-arm manipulation; no serious procedure-related complication or reintervention was recorded in that group.

ACR enables direct visualization but carries risks from surgery and anesthesia. No serious procedure-related complication or reintervention was identified in the ACR group. This does not establish comparative safety: the cohort had limited power for rare harms, and aggregate records did not systematically capture mild or transient events such as pain flares, short-lived sensory symptoms, or recurrent stiffness not requiring reintervention.

### 4.5 Hospital-recorded aggregate treatment amount

The hospital-recorded aggregate treatment amount was lower in the combined-strategy group, but itemized hospitalization, procedure, consumable, medication, and other components were unavailable. The observed difference therefore cannot be explained or interpreted as a standardized direct medical cost.

The amount was observed in a two-center, multiyear setting in which billing systems and prices may differ; site adjustment cannot remove those sources of heterogeneity. Rehabilitation expenses, indirect costs, return to work, and quality-adjusted life-years were not measured. The result is therefore cohort-specific and does not establish cost-effectiveness or generalize directly to other payment systems.

### 4.6 Strengths and limitations

Strengths include a two-center comparison of routine-care strategies, repeated assessments over 12 months, and evaluation of clinical outcomes, serious safety events, and the hospital-recorded aggregate treatment amount. Baseline-adjusted mixed-effects models accounted for within-patient correlation, IPTW assessed the consistency of findings under measured covariate adjustment, and preoperative MRI helped exclude major structural shoulder disease.

The retrospective, nonrandomized design is the main limitation. Shared decision-making may have influenced treatment allocation, and neither regression adjustment nor IPTW can remove unmeasured confounding. Detailed MRI findings, financial circumstances, patient preferences, and surgeon judgment were not consistently available. Excluding 18 patients with shorter follow-up and 8 with insufficient records may also have introduced selection bias; group, site, and baseline characteristics were unavailable for these 26 records. The findings therefore apply to the complete-follow-up cohort. Ridge penalization and truncation stabilized the propensity weights after near-complete separation, but residual confounding remains possible.

The combination treatment prevents attribution of any effect to MUA, PRP, or CS alone. The 1-month VAS contrast was highlighted after data collection and was not prospectively registered, so it should be regarded as exploratory. No study-specific MCID or validated between-group clinical-importance threshold was defined. Outcome assessors were not blinded, and passive-ROM reliability and rehabilitation adherence were not recorded. The reason for missing ACR abduction data was unknown, the missing-at-random assumption could not be tested, and alternative IPTW truncation thresholds were not examined. Surgeon and center effects could not be separated. The procedural summary did not specify whether CS was mixed with PRP or injected separately, or the final injectate volume.

The cohort was too small for precise estimates of rare complications, and mild or transient events were not systematically captured. The recorded aggregate treatment amount lacked itemized components, a defined billing window, and price standardization. It should therefore not be interpreted as a standardized direct medical cost or as evidence of cost-effectiveness.

## 5 Conclusion

In this two-center retrospective cohort, MUA plus PRP and CS was associated with favorable early between-group outcome differences and a lower hospital-recorded aggregate treatment amount than ACR. The 3-month ASES difference was the clearest potentially meaningful early signal. The clinical importance of the 1-month VAS, ASES, and SPADI differences, and of the small passive internal-rotation estimate, remains uncertain. These findings may support shared decision-making, but nonrandom treatment allocation and residual confounding preclude causal or component-specific inferences about faster recovery, comparative safety, equivalence, or cost-effectiveness.

## Data Availability

The raw data supporting the conclusions of this article will be made available by the authors, without undue reservation.

## Supplementary material

SUPPLEMENTARY TABLE S1

Absolute standardized mean differences before and after IPTW

SUPPLEMENTARY TABLE S2

IPTW-weighted longitudinal clinical outcome comparisons

SUPPLEMENTARY FIGURE S1

Covariate balance before and after IPTW
