# Supplementary material for: MUA with PRP and corticosteroid injection versus arthroscopic capsular release for refractory frozen shoulder: a retrospective cohort study
Source: Front Med (Lausanne). 2026 Oct 21;13:1957034. doi: 10.3389/fmed.2026.1957034 (PMC13730863; doi:10.3389/fmed.2026.1957034)
Supplement: Supplementary file 1 [file Supplementaryfile1.docx]

# Supplementary Material

MUA with PRP and corticosteroid injection versus arthroscopic capsular release for refractory frozen shoulder: a retrospective cohort study

**Supplementary Table S1.** Absolute standardized mean differences before and after IPTW.

| **Variable** | **Absolute SMD before IPTW** | **Absolute SMD after IPTW** |
| --- | --- | --- |
| Age | 0.120 | 0.032 |
| Sex | 0.093 | 0.018 |
| Dominant-side involvement | 0.205 | 0.097 |
| Symptom duration | 0.201 | 0.032 |
| Diabetes mellitus | 0.092 | 0.039 |
| Study site | 0.025 | 0.002 |
| Baseline VAS | 0.050 | 0.004 |
| Baseline ASES | 0.292 | 0.084 |
| Baseline SPADI | 0.224 | 0.048 |
| Baseline forward flexion | 0.043 | 0.061 |
| Baseline abduction | 0.070 | 0.084 |
| Baseline external rotation | 0.206 | 0.065 |
| Baseline internal rotation | 0.216 | 0.075 |

Absolute SMD values below 0.10 indicate acceptable covariate balance. IPTW, inverse probability of treatment weighting; SMD, standardized mean difference.

**Supplementary Table S2. IPTW-weighted longitudinal clinical outcome comparisons.**

| **Outcome** | **Follow-up** | **IPTW-weighted MD (Combined - ACR)** | **95% CI** | **Holm-adjusted P value** |
| --- | --- | --- | --- | --- |
| VAS | 1 month | -0.58 | -0.82 to -0.33 | <0.001 |
| VAS | 3 months | 0.14 | -0.06 to 0.34 | 0.467 |
| VAS | 6 months | 0.05 | -0.06 to 0.16 | 0.759 |
| VAS | 12 months | 0.00 | -0.17 to 0.17 | 0.994 |
| ASES | 1 month | 1.90 | 0.71 to 3.08 | 0.005 |
| ASES | 3 months | 9.10 | 7.83 to 10.38 | <0.001 |
| ASES | 6 months | -0.24 | -1.33 to 0.86 | 0.672 |
| ASES | 12 months | -0.41 | -1.24 to 0.41 | 0.645 |
| SPADI | 1 month | -2.72 | -3.70 to -1.74 | <0.001 |
| SPADI | 3 months | 0.02 | -0.36 to 0.41 | 1.000 |
| SPADI | 6 months | -0.03 | -0.43 to 0.36 | 1.000 |
| SPADI | 12 months | 0.08 | -1.00 to 1.15 | 1.000 |
| Forward flexion, degrees | 1 month | 9.49 | 5.22 to 13.77 | <0.001 |
| Forward flexion, degrees | 3 months | 0.39 | -2.48 to 3.27 | 1.000 |
| Forward flexion, degrees | 6 months | -0.91 | -3.06 to 1.23 | 1.000 |
| Forward flexion, degrees | 12 months | -0.01 | -1.52 to 1.49 | 1.000 |
| Abduction, degrees | 1 month | -0.17 | -2.99 to 2.65 | 1.000 |
| Abduction, degrees | 3 months | 0.26 | -2.00 to 2.52 | 1.000 |
| Abduction, degrees | 6 months | 0.69 | -1.77 to 3.14 | 1.000 |
| Abduction, degrees | 12 months | 0.68 | -2.13 to 3.49 | 1.000 |
| External rotation, degrees | 1 month | 0.08 | -1.75 to 1.92 | 1.000 |
| External rotation, degrees | 3 months | -0.00 | -0.99 to 0.98 | 1.000 |
| External rotation, degrees | 6 months | 0.01 | -1.39 to 1.41 | 1.000 |
| External rotation, degrees | 12 months | -0.00 | -1.44 to 1.44 | 1.000 |
| Internal rotation, degrees | 1 month | -0.10 | -1.96 to 1.75 | 1.000 |
| Internal rotation, degrees | 3 months | -0.24 | -1.29 to 0.81 | 1.000 |
| Internal rotation, degrees | 6 months | 0.74 | -0.64 to 2.11 | 0.879 |
| Internal rotation, degrees | 12 months | 2.03 | 0.02 to 4.03 | 0.193 |

IPTW used stabilized weights from a ridge-penalized logistic propensity-score model (L2 penalty parameter, 0.01), truncated at the 2.5th and 97.5th percentiles. The model included age, sex, dominant-side involvement, symptom duration, diabetes mellitus, study site, and all baseline clinical outcomes. Weighted generalized estimating equations with an exchangeable working correlation and robust standard errors estimated postoperative contrasts. MDs are the combined-strategy group minus the ACR group. P values were Holm-adjusted within each outcome across the 1-, 3-, 6-, and 12-month comparisons. Postoperative abduction estimates used 67 combined-strategy and 69 ACR patients. ACR, arthroscopic capsular release; CI, confidence interval; IPTW, inverse probability of treatment weighting; MD, mean difference.

Supplementary Figure S1. Absolute standardized mean differences before and after IPTW. Open circles show balance before weighting, filled circles show balance after weighting, and the dashed line marks the 0.10 threshold. The ridge-penalized logistic propensity-score model included age, sex, dominant-side involvement, symptom duration, diabetes mellitus, study site, and all baseline clinical outcomes. Stabilized weights were truncated at the 2.5th and 97.5th percentiles.
